# Physician attitudes toward natural syrup formulations for cough and sore throat: a multinational cross-sectional survey of 398 healthcare professionals across 13 countries

**DOI:** 10.3389/fphar.2026.1792358

**Published:** 2026-03-02

**Authors:** Barbara LePetri, Cecilia Bartoli, Carolina Castillo

**Affiliations:** 1 Independent Researcher, New York, NY, United States; 2 Medical Affairs, Menarini, Florence, Italy

**Keywords:** glycerol, Malva sylvestris, natural syrup, sorbitol, Verbasci flos

## Abstract

**Background:**

Understanding physician attitudes toward natural over the counter (OTC) products in respiratory medicine remains understudied in multinational contexts. This study represents the first multinational survey of physician perceptions regarding natural syrup formulations for cough and sore throat management.

**Objective:**

To assess physician acceptance of a natural syrup containing Verbascum and Malva sylvestris extracts across diverse international healthcare settings by evaluating seven key product attributes.

**Methods:**

Cross-sectional survey of 398 practicing physicians across 13 countries was conducted through the Sermo global physician network in May 2025. Participants evaluated clinical appropriateness, safety in vulnerable populations, co-prescription capabilities, throat protection properties, allergen-free composition, natural ingredient preference, and nighttime convenience compared to the leading lozenge in each country.

**Results:**

while regional variations were observed, overall physician acceptance was highest for absence of problematic pharmaceutical ingredients (86%), followed by safety in vulnerable populations and co-prescription safety (82%), with lower agreement for natural ingredients (77%), throat protection (73%), allergen-free composition (71%), and night-time convenience (63%). Regional patterns showed consistently higher acceptance in Middle Eastern countries and below-average values in most European countries (except Ireland), with Saudi Arabia highest and Finland and Italy lowest.

**Conclusion:**

Physicians across diverse healthcare systems reported favourable attitudes toward this natural syrup formulation, with particular emphasis on the perceived safety profile for vulnerable patient groups. The notable international variation suggests the potential influence of cultural and regional factors on prescribing attitudes. While these exploratory findings should be interpreted cautiously given the descriptive study design and industry sponsorship, they provide preliminary support for the continued investigation of natural formulations in respiratory care, particularly for patients with comorbidities who may require alternatives to conventional antitussive therapies.

## Introduction

Natural products have gained increasing recognition in respiratory healthcare as viable alternatives or complementary treatments to conventional pharmaceutical interventions (Oriola and Oyedeji, 2022). The development of natural syrups for respiratory conditions draws from centuries-old traditional medicine practices, now supported by modern scientific validation (Wolf et al., 2023). These formulations combine the therapeutic benefits of medicinal plants with biocompatible excipients to create effective, well-tolerated treatments for various respiratory ailments (Berkenfeld et al., 2024).

The formulation of natural respiratory syrups requires careful consideration of both active botanical components and functional excipients that contribute to the product’s stability, palatability, and therapeutic efficacy. The botanical components in natural respiratory syrups are selected based on extensive ethnobotanical knowledge and emerging scientific evidence. *Verbascum* species, have been traditionally used in European folk medicine for treating respiratory conditions including cough, bronchitis, and upper respiratory tract infections (Blanco-Salas et al., 2021). It is the largest genus of the family, and its origin is the centre of the Eastern Mediterranean Basin (Blanco-Salas et al., 2021). Recent phytochemical analyses have identified numerous bioactive compounds in *Verbascum* species. Their bioactive compounds: flavonoids, phenolic acids, and iridoid glycosides, exhibit anti-inflammatory, antioxidant, and antimicrobial properties that support respiratory mucosal health (Garcia-O et al., 2022; Morote et al., 2024).

*Malva sylvestris* represents another cornerstone of traditional respiratory medicine, recognised for its mucilaginous properties and anti-inflammatory activities. The *M. sylvestris* is an annual herb with shallowly lobed leaves and purple flowers blooming at late spring (Elsagh et al., 2015). The plant is rich in mucilaginous polysaccharides, flavonoids, and phenolic derivatives, exerts demulcent and anti-inflammatory effects. Clinical evidence demonstrates reductions in both daytime and nighttime cough scores in children treated with *M. sylvestris*-based syrups. The mucilage content, comprising 6%–8% of the leaves, consists of high-molecular weight acidic polysaccharides that provide demulcent and soothing effects on irritated respiratory mucosa (Batiha et al., 2023; Carnevali et al., 2021; Mousavi et al., 2021).

Modern analytical techniques have revealed that the therapeutic efficacy of natural syrups extends beyond their active botanical components (Dzobo, 2022; Srivastava et al., 2013; Yang et al., 2013). Importantly, excipients in these formulations serve more than structural purposes; they actively contribute to symptom relief through demulcent, lubricating, and soothing actions (Berkenfeld et al., 2024). Glycerol, for example, functions as a humectant and demulcent, forming a protective layer over irritated mucous membranes (Eccles, 2020; Eccles and Mallefet, 2017). Similarly, sorbitol provides sweetening properties with fewer calories than natural sugars while contributing to the syrup’s stability and palatability (Eccles, 2020). The synergistic effects of combining multiple botanical extracts in a single formulation may enhance therapeutic outcomes through complementary mechanisms of action. The total composition, including excipients, contributes to the overall therapeutic effect through various mechanisms including mechanical barrier formation, moisture retention, and enhanced bioavailability of active compounds. The incorporation of functional excipients like hydroxypropyl methylcellulose and xanthan gum further enhances the product’s demulcent properties by increasing viscosity and residence time on mucosal surfaces (Eccles, 2020; Eccles and Mallefet, 2017).

Given that conventional cough and cold medications may be contraindicated in patients with comorbidities or polypharmacy, natural formulations provide valuable alternatives with fewer side effects and better tolerability. Physicians often avoid prescribing dextromethorphan in patients with asthma or chronic obstructive pulmonary disease (COPD), as well as cough syrups containing sugar in patients with diabetes, and generally agree that natural products may represent an appropriate option for individuals with comorbidities, given that many conventional cough and cold remedies can interact with concomitant medications or exacerbate chronic disease symptoms. Moreover, natural formulations are increasingly viewed as suitable alternatives for patients who are sensitive to the side effects of standard therapies, particularly when evidence from clinical studies supports their safety and efficacy (Canonica et al., 2025). Understanding physician attitudes toward natural OTC products represents a critical gap in respiratory medicine research, particularly regarding the factors that influence treatment selection for patients with cough and respiratory symptoms. The objective of the present study was to systematically assess physician perceptions of a natural syrup formulation designed for irritating cough and sore throat in the context of common upper respiratory conditions including acute bronchitis, pharyngitis and laryngitis across multiple international healthcare settings by determining physician acceptance of seven key product attributes.

## Methods

### Design and participants

The methodology for this study has been comprehensively described in a previous publication (Canonica et al., 2025). Briefly, this multinational cross-sectional survey was conducted in May 2025 among 398 practicing physicians across 13 countries using established online survey protocols through the Sermo global physician network. Eligible participants were practicing physicians spending ≥65% of their clinical time in active patient care, who voluntarily participated in this anonymous market research study with compensation provided. The 13 participating countries (Colombia, Finland, France, Ireland, Italy, Lithuania, Peru, Portugal, Saudi Arabia, South Africa, Spain, United Arab Emirates, and United Kingdom) were selected based on product market availability, representation of diverse healthcare systems (European, Middle Eastern, Latin American, and African), and coverage within the Sermo physician network. Participating physician specialties included general practitioners (46%), specialists (42%), and family practitioners (12%), as detailed in the primary publication (Canonica et al., 2025).

The current study evaluates physician perceptions, acceptance, and clinical reasoning related to the use of a natural syrup formulation for managing irritating cough and sore throat (named Product X in the questionnaire). This triple-acting syrup formulation contains natural botanical extracts within a hydrocolloid matrix comprising purified water (72.17% w/w), glycerol E 422 (15.26% w/w), sorbitol E 420i (10.18% w/w), *Verbasci flos* extract (0.78% w/w), hydroxy-propyl methyl cellulose E 464 (0.60% w/w), *M. sylvestris* leaf extract (0.46% w/w), xanthan gum E 415 (0.21% w/w), potassium sorbate E 202 (0.18% w/w), and citric acid E 330 (0.15% w/w) (hereafter referred to as the evaluated formulation). Participants reviewed standardised product profile and completed structured evaluation using a structured questionnaire. The survey instrument was developed through an iterative process involving content review by clinical experts in respiratory medicine and phytopharmacology to ensure face validity and content relevance. Item construction followed standard principles for attitudinal survey design and employed Likert-type response scales anchored to each statement. Although psychometric validation was not conducted for this instrument, the questionnaire items were derived from clinically relevant product attributes identified through prior literature review and expert consultation (Canonica et al., 2025). The survey items were designed as attitudinal probes reflecting the product’s label claims, and respondents were evaluating their level of agreement with these manufacturer-defined attributes.

### Product device attributes analysis

Physician assessment focused on seven key attributes of the natural syrup formulation, each designed to capture distinct clinical and patient-centred considerations relevant to respiratory symptom management. These attributes included (Oriola and Oyedeji, 2022): clinical appropriateness through exclusion of alcohol, codeine, caffeine, and paracetamol/non-steroidal anti-inflammatory drugs (NSAIDs) (Wolf et al., 2023); safety and effectiveness in vulnerable populations including patients with asthma, COPD, COVID-19, and geriatric individuals with persistent cough (Berkenfeld et al., 2024); safe co-prescription capabilities without drug interaction risks associated with pseudoephedrine or dextromethorphan (Blanco-Salas et al., 2021); throat protection against bacterial and viral pathogens (Garcia-O et al., 2022); patient appeal via absence of gluten, sugar, lactose, and flavour additives (Morote et al., 2024); patient preference for natural ingredients based on the botanical extract composition; and (Elsagh et al., 2015) convenience advantages over traditional lozenges for night-time cough management.

### Statistical analyses

Descriptive statistics characterised physician evaluation patterns for the natural syrup formulation. Country-specific analyses examined international variations in product assessment scores. Response distributions were calculated for Likert-scale items measuring product attribute importance ratings, with detailed analytical methodology available in the primary publication (Canonica et al., 2025). Given the exploratory nature of this survey and the sample size of approximately 30 physicians per country, inferential statistical testing was not applied to between-country comparisons, as the study was not powered for formal hypothesis testing at the national level. Accordingly, all cross-national findings should be interpreted as exploratory and hypothesis-generating rather than confirmatory.

## Results

**FIGURE 1:**
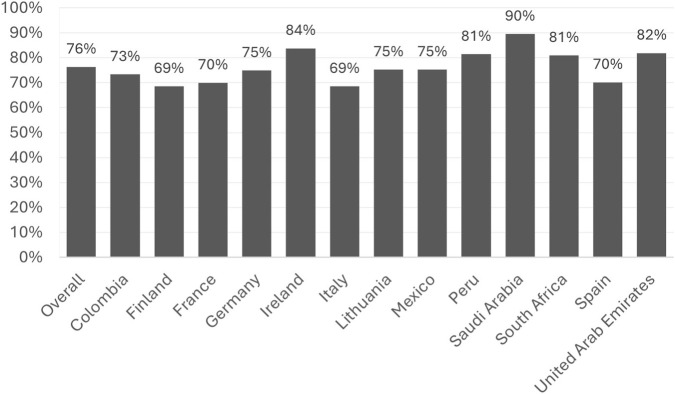
Average results for the seven product attributes by country.

A total of 398 physicians across the 13 countries (30 physicians per country, with the exception of Germany [33] and Spain [35]) evaluated seven key attributes of the natural syrup formulation. Physician specialties and country of clinical practice have been described elsewhere (Canonica et al., 2025). Overall results for all interrogated attributes are presented in Table 1. Average results for the seven attributes demonstrated that physicians reported overall favourable attitudes toward the natural composition of Product X across multiple patient populations, with a mean acceptance rate of 76% (Figure 1).

**TABLE 1:** Overall results for the seven interrogated attributes by question.

Question	Overall
Product X does not contain alcohol, codeine, caffeine, or paracetamol/NSAIDs, which makes it appropriate for many types of patients	86%
Product X can be safely co-prescribed along with other cough/cold medications, unlike medications containing pseudoephedrine or dextromethorphan	82%
Product X is a safe and effective choice for asthma, COPD, COVID, and geriatric patients with persistent cough	82%
Patients will like that product X contains only natural ingredients	77%
Product X protects the throat from bacteria and viruses	73%
Product X does not contain gluten, sugar, lactose, or flavour additives, which makes it appealing to many types of patients	71%
For patients troubled by night-time cough, product X is more convenient than taking a lozenge at bed-time	63%

When specifically analyzing the product attributes, a large majority (86%) recognized its absence of alcohol, codeine, caffeine, and paracetamol/NSAIDs as making it appropriate for diverse patients, while 71% noted the lack of gluten, sugar, lactose, and flavor additives increased its appeal. Importantly, 82% agreed that Product X can be safely co-prescribed with other cough and cold medications, unlike products containing pseudoephedrine or dextromethorphan. Physicians also emphasized patient-centric benefits, with 77% indicating that patients would value its all-natural formulation and 63% finding it more convenient for night-time cough management compared with lozenges. Furthermore, 82% considered Product X a safe and effective choice for patients with asthma, COPD, COVID-19, or in geriatric populations, while 73% agreed it helps protect the throat from bacterial and viral irritation.

### Regional variations

In almost all countries (except Finland and Italy), the positive impression, when combining the average results per country for the seven attributes analyzed, was 70% or higher. All European countries, except for Ireland, show acceptance values below the overall results, while middle east countries show above the overall value (Figure 1). There is no homogeneity in the average values from Latin American countries. Saudi Arabia is the country that shows the highest physician acceptance, and Finland and Italy show the lowest acceptance.

Regarding the absence of problematic ingredients, South Africa demonstrated a 100% physician agreement, while Spain showed the lowest agreement (60%), representing the largest inter-country variation observed across all attributes. Latin American countries (Colombia 97%, Peru 97%) showed a very strong agreement with this attribute (Supplementary Figure S1).

Peru, Saudi Arabia, South Africa, and the United Arab Emirates demonstrated the highest acceptance rates at 90% regarding the product’s suitability for vulnerable patient populations, including those with asthma, COPD, COVID-19, and geriatric patients presenting with persistent cough, while France exhibited the lowest physician acceptance at 70% (Supplementary Figure S2). Regarding co-prescription safety, the United Arab Emirates achieved the highest physician agreement at 97%, whereas Italy showed the most conservative response at 63%. Among European nations, Lithuania and Ireland both demonstrated strong appreciation for co-prescription safety at 93% acceptance rates (Supplementary Figure S3).

Regarding country specific results for throat protection against bacterial and viral pathogens, Saudi Arabia achieved the highest physician endorsement with 93% physician agreement, while only 50% of agreement among French physicians (Supplementary Figure S4).

The absence of common allergens and additives, was particularly valued among Saudi Arabian physicians, with 87% approval, while French physicians demonstrated comparatively lower appreciation for this characteristic at 53% acceptance (Supplementary Figure S5).

When evaluating the attribute of only containing natural ingredients, the most between countries remarkable variation was observed between Ireland (97%) and Finland (67%). Middle Eastern countries showed consistently high appreciation for natural ingredients, with UAE (70%) being the only exception in this region (Supplementary Figure S6).

The night-time convenience advantage Saudi Arabia led physician endorsement at 83%, representing the strongest belief in the product’s night-time convenience advantage. This was followed by Ireland at 77%, indicating substantial physician confidence in this comparative claim. The most reserved responses came from Lithuania (50%), Italy and South Africa (both 53%) (Supplementary Figure S7).

## Discussion

To our knowledge, this is the first study to evaluate physician preferences for a natural syrup formulation containing botanical extracts (Verbascum and Malva sylvestris) within a hydrocolloid matrix, designed for the symptomatic management of cough and sore throat, particularly in the context of common upper respiratory conditions such as acute bronchitis, pharyngitis, and laryngitis. The product’s mechanism of action relies primarily on the formation of a mucoadhesive film that adheres to the pharyngeal and laryngeal mucosa, with the botanical extracts contributing complementary anti-inflammatory and demulcent properties. This multinational survey of 398 physicians across 13 countries provides preliminary evidence of favourable physician perceptions toward this natural upper respiratory formulation, particularly regarding its safety profile and perceived appropriateness for vulnerable patient populations. The findings offer exploratory insights into international variations in clinical decision-making and highlight the evolving role of natural syrups in contemporary respiratory medicine.

The high physician endorsement of formulations lacking problematic pharmaceutical ingredients is consistent with growing clinical interest insafer therapeutic alternatives, though the observed inter-country variability suggests that cultural and regulatory factors also influence prescribing attitudes. This observation aligns with growing clinical concerns about the adverse effects associated with traditional cough suppressants, particularly codeine-related respiratory depression in paediatric populations and cardiovascular risks associated with pseudoephedrine in elderly patients (Kernan et al., 2000).

Additionally, the strong physician recognition of safety in vulnerable populations reflects contemporary clinical priorities and administration recommendations, particularly relevant given the increasing prevalence of respiratory comorbidities in ageing populations and the ongoing management of post-COVID respiratory symptoms (Joshi, 2024). The formulation’s glycerol and sorbitol-based matrix, combined with natural botanical extracts, addresses these clinical needs by providing symptomatic relief (Jacob et al., 2023) without the contraindications associated with conventional antitussives (Berkenfeld et al., 2024). The formulation’s clinical advantage also lies in its sugar-free and alcohol-free composition, which makes it particularly suitable for patients with compromised health status, including diabetic individuals, paediatric populations, geriatric patients, and those with comorbidities requiring dietary restrictions. These findings are consistent with current clinical and regulatory guidance emphasizing the avoidance of potentially harmful ingredients in symptomatic cough treatments. The American College of Chest Physicians recommends against the use of codeine-containing medications in pediatric patients because of the risk of severe adverse effects, and discourages the use of nonsteroidal anti-inflammatory drugs for cough management until clinical efficacy is demonstrated (Chang and Glomb, 2006). Similarly, the U.S. Food and Drug Administration has issued restrictions and safety warnings regarding codeine use in children and the concomitant administration of opioids and central nervous system depressants (FDA Drug Safety Communication, 2023).

The moderate-to-strong physician acceptance of throat protection claims suggests growing professional recognition of the antimicrobial and anti-inflammatory properties attributed to *Verbascum* and *M. sylvestris* extracts (Batiha et al., 2023; El et al., 2017). Phytochemical research provides a plausible mechanistic basis for these attitudinal observations, demonstrating significant bioactive compound concentrations in both botanical components, including flavonoids, phenolic acids, and mucilaginous compounds that contribute to mucosal protection and anti-inflammatory activity (Batiha et al., 2023). The mucoadhesive film formation represents the core therapeutic mechanism, creating a protective barrier over inflamed or irritated mucosal surfaces. This biocompatible film provides sustained contact time with the affected tissue, potentially enhancing therapeutic efficacy compared to conventional liquid preparations that may have limited residence time in the oral cavity. The pronounced variation between Saudi Arabian (93%) and French (50%) physician acceptance may reflect different clinical experiences with botanical medicines and varying integration of traditional medicine concepts into modern healthcare practice.

The physician preference for allergen-free formulations reflects increasing awareness of dietary restrictions and food sensitivities in clinical practice and is consistent with current clinical guidance emphasizing harm reduction and individualized treatment selection in patients at elevated risk of drug-related adverse effects. A distinguishing feature of this formulation compared with some traditional natural throat remedies is its reliance on a hydrocolloid matrix (hydroxypropyl methylcellulose and xanthan gum) as the primary mucoadhesive vehicle, rather than honey or high-concentration plant extracts, which may carry allergenic potential. The botanical extracts (Verbascum and Malva sylvestris) are present at relatively low concentrations (0.78% and 0.46% w/w, respectively), contributing complementary bioactive properties within the mucoadhesive framework. The exclusion of gluten, sugar, lactose, and artificial additives from the natural syrup formulation addresses these clinical concerns while maintaining therapeutic efficacy. Additionally, populations such as those with asthma, COPD, or advanced age often require therapeutic approaches with minimal systemic exposure or potential for pharmacologic interactions. These results reflect physicians’ adherence to safety-driven principles highlighted by both respiratory and geriatric practice guidelines (Chang and Glomb, 2006; Rajvanshi et al., 2024), which recommend avoiding ingredients capable of triggering intolerance, hypersensitivity reactions, or worsening airway reactivity.

Several methodological limitations should be acknowledged when interpreting these findings. First, the web-based survey approach, although enabling broad international participation, may have introduced selection bias toward physicians who are comfortable with digital technology and have reliable internet access. The English-language requirement may have excluded healthcare professionals with limited English proficiency, potentially underrepresenting clinical perspectives from non-English speaking regions. Second, the sample size of approximately 30 participants per country constrains the statistical power for between-country comparisons; accordingly, all cross-national patterns reported herein should be regarded as exploratory observations rather than definitive conclusions. Third, volunteer bias represents a plausible concern, as physicians with specific interest in respiratory therapeutics or natural products may have been more likely to participate compared to the general medical population. Fourth, the distribution of physician specialties across participating countries may have introduced additional variability, as clinical attitudes toward natural products can differ substantially between generalists and specialists; the specialty composition of the sample has been described in detail in the primary publication (Canonica et al., 2025). Fifth, the 13 countries included in this study were selected based on product availability and Sermo network representation, and do not represent all regions within the Sermo global physician network; therefore, generalisability to unrepresented healthcare systems remains limited. Sixth, the survey instrument, while developed through expert consultation and iterative review, did not undergo formal psychometric validation which limits confidence in the measurement properties of the tool. Seventh, the descriptive analytical approach, although appropriate for this exploratory cross-sectional design, does not permit causal inferences or adjustment for potential confounders such as physician age, years of experience, or prior familiarity with the product. Finally, as an industry-sponsored study, the potential for bias in question framing and interpretation warrants careful consideration, notwithstanding the measures implemented to ensure methodological independence as described in the conflict of interest section. In this regard, several survey items were formulated using affirmative or positively framed statement, which may have introduced acquiescence bias or social desirability effects, thereby potentially inflating agreement rates beyond what would be obtained with neutrally phrased items or balanced response formats. Readers should therefore interpret the reported acceptance percentages in the context of this item framing, and future research should employ validated instruments with counterbalanced or neutrally worded items to minimise such methodological artefacts. Despite these methodological limitations, the study demonstrates several notable strengths. The multinational design successfully captured clinical perspectives from diverse healthcare systems, providing valuable insights into cross-cultural differences in therapeutic approaches and clinical decision-making patterns. The high response rate and broad specialty representation enhance the external validity of our findings across different clinical practice environments. The detailed evaluation of product characteristics offers specific insights into the features that practicing physicians consider most important, which has direct implications for future product development and clinical guideline formulation. The combination of strong response rates and diverse specialty participation further supports the generalizability of these findings to various healthcare settings and patient populations.

## Conclusion

This exploratory study represents the first multinational assessment of physician attitudes toward a natural syrup medical device designed for cough and sore throat pain management through mucoadhesive film formation, revealing remarkable professional consensus across diverse healthcare systems. The survey of 398 physicians spanning 13 countries indicates generally favourable perceptions of the formulation’s safety profile and perceived suitability for diverse patient populations, including those with respiratory comorbidities, though with notable regional variation. Future research should prioritise randomised controlled trials evaluating clinical efficacy and patient-reported outcomes between this natural syrup formulation and conventional antitussive treatments. It is important to recognise that the findings of this survey capture physician attitudes and perceptions rather than clinical evidence of efficacy, pharmacological activity, or safety. The agreement rates reported here reflect professional opinions expressed in response to a standardised product description, and therefore should not be equated with clinical outcomes derived from controlled therapeutic interventions or used to support claims regarding the product’s safety or efficacy in any patient population.

*The following product description was presented verbatim to survey respondents as part of the survey instrument:* Product X is a triple-acting syrup for cough and sore throat that is formulated with a combination of proven natural extracts. It creates a protective film that coats and hydrates the throat, calms the cough, and helps clear mucus, protects the throat from bacteria, viruses, and environmental irritants, it is alcohol-free, codeine-free, caffeine-free, and NSAIDS/analgesics-free, and it is also gluten-free, sugar-free, lactose-free, and free of flavour additives. Product X can be used in adults and children 3+.

## Data Availability

The original contributions presented in the study are included in the article/Supplementary Material, further inquiries can be directed to the corresponding author.
