# Supplementary material for: Physician attitudes toward natural syrup formulations for cough and sore throat: a multinational cross-sectional survey of 398 healthcare professionals across 13 countries
Source: Front Pharmacol. 2026 Mar 2;17:1792358. doi: 10.3389/fphar.2026.1792358 (PMC12989759; doi:10.3389/fphar.2026.1792358)
Supplement: Supplementary file 1 [file Supplementaryfile1.docx]

**Supplementary Figure 1.** Product X does not contain alcohol, codeine, caffeine, or paracetamol/NSAIDs, which makes it appropriate for many types of patients.

**Supplementary Figure 2.** Product X is a safe and effective choice for asthma, COPD, COVID, and geriatric patients with persistent cough.

**Supplementary Figure 3.** Product X can be safely co-prescribed along with other cough/cold medications, unlike medications containing pseudoephedrine or dextromethorphan.

**Supplementary Figure 4.** Product X protects the throat from bacteria and viruses.

**Supplementary Figure 5.** Product X does not contain gluten, sugar, lactose, or flavored additives, which makes it appealing to many types of patients.

**Supplementary Figure 6.** Patients will like that Product X contains only natural ingredients.

**Supplementary Figure 7.** For patients troubled by night-time cough, Product X is more convenient than taking a lozenge at bed-time.
